# How to visually diagnose mechanical dyssynchrony in cardiac resynchronization therapy candidates using echocardiography

**DOI:** 10.1093/ehjimp/qyaf158

**Published:** 2025-12-20

**Authors:** Pedro G Diogo, Kenji Demesure, Alexis Puvrez, Gábor Vörös, Jürgen Duchenne, Jens-Uwe Voigt

**Affiliations:** Department of Cardiovascular Sciences, KU Leuven, Herestraat 49, Leuven 3000, Belgium; Department of Cardiovascular Sciences, KU Leuven, Herestraat 49, Leuven 3000, Belgium; Department of Cardiovascular Sciences, KU Leuven, Herestraat 49, Leuven 3000, Belgium; Centre for Heart Rhythm Disorders, University of Adelaide, The Royal Adelaide Hospital Cardiology, Adelaide, SA, Australia; Department of Cardiovascular Sciences, KU Leuven, Herestraat 49, Leuven 3000, Belgium; Department of Cardiovascular Diseases, University Hospitals Leuven, Leuven, Belgium; Department of Cardiovascular Sciences, KU Leuven, Herestraat 49, Leuven 3000, Belgium; Department of Cardiovascular Diseases, University Hospitals Leuven, Leuven, Belgium; Research Group Cardio & Organ Systems, UHasselt, Diepenbeek, Belgium; Department of Cardiology, Jessa Hospital, Hasselt, Belgium; Department of Cardiovascular Sciences, KU Leuven, Herestraat 49, Leuven 3000, Belgium; Department of Cardiovascular Diseases, University Hospitals Leuven, Leuven, Belgium

**Keywords:** cardiac resynchronization therapy, left bundle branch block, mechanical dyssynchrony, echocardiography, septal flash, apical rocking

## Abstract

Cardiac resynchronization therapy (CRT) improves outcomes in patients with heart failure and broad QRS complex, yet 20–45% do not respond. Mechanical dyssynchrony (MechDys)—identified visually by septal flash and/or apical rocking (SFoAR)—is strongly associated with CRT benefit. This ‘How to’ paper outlines a practical four-step workflow for the visual assessment of MechDys. First, a high-quality, multi-view echocardiographic acquisition is essential. Second, septal flash4 (SF) is recognized as an early leftward septal motion, often with rebound, preceding lateral wall contraction; its magnitude depends on conduction delay, myocardial contractility, and right heart loading. Third, ApR is identified as a biphasic apical motion reflecting sequential septal and lateral wall contractions; its appearance may be modified by scarring, pacing, or imaging artefacts. MechDys is confirmed when either motion pattern is present. Clinically, the visual assessment of MechDys may improve patient selection for CRT, thus improving response rates. The ongoing AMEND-CRT trial is evaluating whether incorporating SFoAR assessment is non-inferior to guideline recommendations. Pending prospective evidence, existing observational data supports the use of visual assessment of MechDys to guide decision-making in patients with intermediate CRT indications.

## Introduction

Cardiac resynchronization therapy (CRT) is an established device therapy for patients with heart failure with reduced ejection fraction and a broad QRS complex on surface electrocardiogram.^1^ CRT has been shown to significantly improve cardiac function and quality of life, and reduce morbidity and mortality. However, not all patients derive equal benefit from CRT, with non-response reported in 20–45% of cases1. Current guidelines suggest that patients with a broad QRS complex with left bundle branch block (LBBB) morphology derive the most benefit, while patients with a narrower LBBB or non-LBBB benefit less. Selecting the right patients for this therapy is therefore a challenging task.

Indeed, although delayed LV electrical conduction is the underlying cause, the pathophysiological remodelling and eventually heart failure in these patients is rather driven by the resulting dyssynchronous mechanical activation of the LV and the subsequent imbalance in regional myocardial loading. There is a significant body of evidence suggesting that this mechanical dyssynchrony (MechDys)—a specific septal-to-lateral contraction pattern of the left ventricle (LV)—is the actual substrate amenable by CRT.^2^ Importantly, the surface QRS does not fully reflect this pattern: not all patients with broad QRS show MechDys, especially those with non-LBBB morphologies who are often non-dyssynchronous. A precise definition of MechDys and reliable methodologies to identify it, would therefore be useful to better select patients that may benefit from CRT.

Earlier studies—using Pulsed Wave and Tissue Doppler—suggested that echocardiographic assessment of LV dyssynchrony was of limited value to predict CRT response.^3^ However, contemporary studies indicate that the detection of other markers of MechDys is strongly associated with both volumetric response and lower mortality after CRT.^2^ Of note, MechDys induced by chronic right ventricle (RV) pacing is likewise predictive of CRT response.^4^

The visual detection of MechDys relies on the presence of SF^5^ and/or apical rocking^6^ (ApRock), collectively abbreviated as septal flash and/or apical rocking (SFoAR). Previous research has described high expert intra- and inter-observer agreement for the detection of these motion patterns.^2,4^

**Figure 1:**
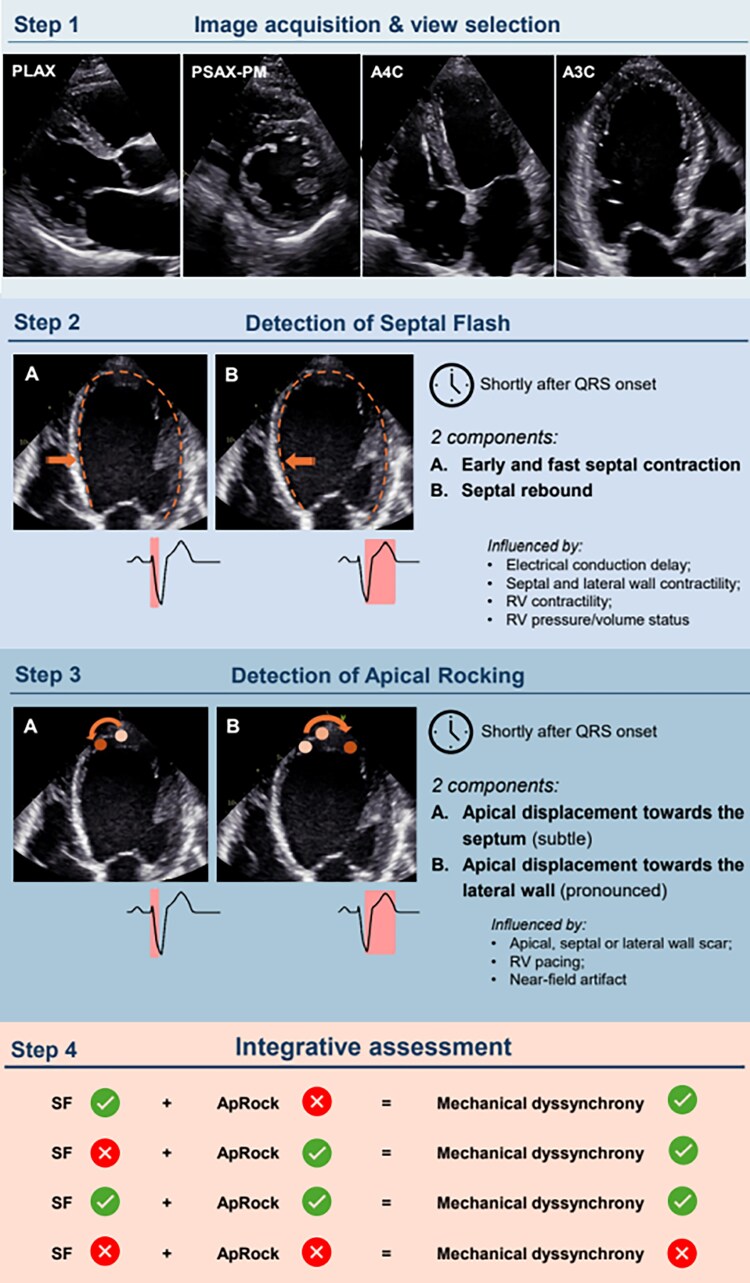
Four steps for diagnosing mechanical dyssynchrony in CRT candidates using echocardiography. A4C, apical 4-chamber view; A3C, apical 3-chamber view; ApRock, apical rocking; PLAX, parasternal long-axis; PSAX, parasternal short-axis; SF, septal flash; RV, right ventricle.

This ‘How To’ article aims to provide a practical guidance to recognize these motion patterns and perform a visual echocardiographic assessment of MechDys in CRT candidates (*Figure 1*).

## Step 1: image acquisition and view selection

While detecting the presence of pronounced SFoAR is often straight-forward and immediate, even in poor-quality exams, confirming the absence of SFoAR requires high-quality echocardiographic images as the characteristic patterns can also be subtle. Standard parasternal, subcostal and focused apical 2, 3- and 4-chamber views are sufficient. Care should be taken to avoid image motion due to breathing as well as premature beats. Also, adequate electrocardiogram (ECG) gating is crucial to avoid jumping image loops. As SFoAR often begin just around the ECG trigger point, the acquisition of three cardiac cycles is advised to have at least two uninterrupted transitions among cycles. Because SFoAR can be very subtle, slowing down the clips or even performing a frame-by-frame analysis may be necessary for decision-making. Also, patients with frequent ectopic beats or irregular rhythms mandate a more thorough assessment.

## Step 2: detection of SF

SF is caused by the early contraction of the interventricular septum against the still low LV pressure, and is the first mechanical event in the dyssynchronous LV.^7^ SF is defined by the following elements:

1. SF is a fast and short-lived event that occurs shortly after the onset of the QRS complex;
2. SF comprises an early contraction of the septum that results in its leftward motion (towards the left ventricle), and is often followed by a visible rebound motion in the opposite direction (towards the right ventricle);
3. The septal contraction precedes the delayed contraction of the lateral wall.

SF can range from a very clear sequence of leftward and rightward (rebound) motions to a minimal leftward motion without a perceptible rebound. Also, SF may occur in almost the entire septum or can be localized to a single septal segment. SF is typically seen in apical 4- and 3-chamber views but may in some cases only be visible in parasternal or subcostal views, which highlights the importance of examining the entire septum from multiple echocardiographic views.

Several factors might influence the magnitude of SF^7^:

- Electrical conduction delay. QRS duration reflects the right ventricular-to-left ventricular and LV septal-to-lateral activation delays: a broader QRS with greater conduction delay typically results in a more prominent SF;
- Septal contractility. Reduced septal contractility, due to disease-related remodelling or septal scar, attenuates SF;
- RV contractility and pressure/volume status. Reduced RV systolic function can also attenuate SF. Elevated RV pressure or RV volume overload typically displace the septum leftward, a motion that can both mimic SF and reduce its magnitude when present.
- Lateral wall contractility. Presence of lateral wall scarring also attenuates SF by suppressing the septal rebound motion;

## Step 3: detection of ApRock

ApRock refers to a specific motion sequence of the apex, with an initial lateral-to-septal, and later septal-to-lateral components, perpendicular to the LV long axis. This ‘rocking’ motion is caused by the sequential contraction of the septum and, later, the lateral wall. It can be recognized by:

1. A short-lived and subtle displacement of the apex towards the septum, occurring shortly after the onset of the QRS complex, which reflects the pulling of the contracting septum during SF;
2. A subsequent longer, more pronounced lateral displacement of the apex. This second phase corresponds to the pulling effect of the contracting lateral wall. It is a systolic event and therefore starts before mitral valve opening.

Any other motion pattern should not be considered ApRock, e.g. an inverted sequence with a septal-to-lateral motion first or the lateral apical pulling by the LV found when dilated RVs become apex-forming. Like for SF, the magnitude of ApRock is influenced by different factors:

- Scarring. Apical scar can attenuate ApRock; additionally, both septal and lateral wall scarring can also reduce the magnitude of ApRock, as their contractility is necessary to produce the ‘rocking’ motion sequence;
- RV pacing. Apical septal stimulation might lead to a more prominent septal displacement and a more attenuated lateral motion, resulting in an atypical ApRock pattern;
- Near-field artefact can reduce the visibility of the apex, influencing the detection of subtle ApRock. Foreshortening, on the other hand, may amplify the appearance of ApRock but can also limit its clear distinction from other contraction patterns.

## Step 4: integrative assessment

The presence of either motion pattern (SF and/or ApRock) in CRT candidates indicates the presence of MechDys. Since both patterns are closely linked—the first phase of ApRock results from the pulling effect of SF—both patterns can typically be identified in a majority of dyssynchronous patients. However, in some cases only one of the two patterns is detected, due to either patient-specific factors or technical limitations as mentioned above. While this ‘How To’ article focuses on the visual assessment of MechDys, also M-mode and speckle-tracking-imaging^8,9^ (septal strain curve analysis, systolic stretch index or lateral-to-septal work difference) may be used. While their application may be of help for the less experienced reader, they have not shown to increase accuracy of an expert and their detailed description goes beyond the scope of the current manuscript.

## Clinical relevance

AMEND-CRT (NCT04225520) is an ongoing randomized controlled trial testing the hypothesis that incorporating the visual assessment of MechDys into current CRT selection criteria is non-inferior to current guideline-based recommendations.^10^ A positive outcome of this RCT would provide strong evidence for integrating visual assessment of MechDys into current recommendations and routine clinical practice. While we await robust prospective evidence, existing observational studies^2^ already suggest that visual assessment of MechDys can be considered to support decision-making in patients with Class II indication for CRT.

## Data Availability

No new data were generated or analysed in support of this research.

## Lead author biography

Pedro Grilo Diogo is a Portuguese cardiologist currently completing a PhD in Cardiovascular Sciences at the Catholic University of Leuven (Belgium), focusing on the assessment of mechanical dyssynchrony and response to CRT. He also holds a master’s degree in Healthcare Professionals Education from Maastricht University (The Netherlands). Dr Diogo previously trained as a Fellow at the University Hospital of São João (Porto, Portugal), where he developed his clinical interests in echocardiography and heart failure. He is passionate about integrating echocardiography into daily clinical practice and is committed to improving patient care through the education of healthcare professionals.
